# Complete Genome Sequence of an Avian Metapneumovirus Subtype A Strain Isolated from Chicken (*Gallus gallus*) in Brazil

**DOI:** 10.1128/genomeA.00688-17

**Published:** 2017-07-20

**Authors:** Laís S. Rizotto, Guilherme P. Scagion, Tereza C. Cardoso, Raphael M. Simão, Leonardo C. Caserta, Julia C. Benassi, Lara B. Keid, Trícia M. F. de S. Oliveira, Rodrigo M. Soares, Clarice W. Arns, Steven Van Borm, Helena L. Ferreira

**Affiliations:** a Department of Veterinary Medicine, Faculty of Animal Science and Food Engineering, University of Sao Paulo (FZEA-USP), Pirassununga, Brazil; b Graduate Program in Experimental Epidemiology of Zoonoses, Faculty of Veterinary Medicine and Animal Science, University of Sao Paulo (FMVZ-USP), Sao Paulo, Brazil; c Molecular Platform Unit, CODA-CERVA Veterinary and Agrochemical Research Centre, Brussels, Belgium; d DAPSA Department, Laboratory of Animal Virology and Cell Culture, College of Veterinary Medicine, Universidade Estadual Paulista, Araçatuba, Sao Paulo, Brazil; e Laboratory of Animal Virology, Institute of Biology, University of Campinas-UNICAMP, Campinas, Brazil

## Abstract

We report here the complete genome sequence of an avian metapneumovirus (aMPV) isolated from a tracheal tissue sample of a commercial layer flock. The complete genome sequence of aMPV-A/chicken/Brazil-SP/669/2003 was obtained using MiSeq (Illumina, Inc.) sequencing. Phylogenetic analysis of the complete genome classified the isolate as avian metapneumovirus subtype A.

## GENOME ANNOUNCEMENT

Avian metapneumovirus (family *Pneumoviridae*, genus *Metapneumovirus*) (1) is the viral agent responsible for rhinotracheitis of turkeys and is associated with swollen head syndrome in chickens (2, 3). The tracheal tissue sample used in this study was obtained from a Brazilian commercial layer flock showing swollen head syndrome, previously detected by reverse transcription (RT)-PCR (4) and confirmed by real-time RT-PCR (5). Samples were collected according to international, national, and institutional guidelines for the care and use of animals.

The virus was isolated in chicken embryo-related cells after three passages. The cell supernatant sample was pretreated according to a previous study (6). Afterward, RNA extraction was performed with TRIzol (Ambion, Thermo Fisher Scientific) and an RNeasy MinElute clean-up kit (Qiagen). The RNAs were quantified by spectrophotometry and Qubit (Invitrogen) fluorimetry. Both cDNA synthesis and double-stranded DNA synthesis were performed using the SuperScript IV first-strand cDNA synthesis (Invitrogen) and second-strand synthesis mRNA kits (New England Biolabs), respectively, following the manufacturers’ instructions using random hexamer primers. The libraries were prepared with the Nextera XT sample preparation kit (Illumina, Inc.) using standard Nextera XT adapters for library multiplexing. The quantification of the libraries was performed with the SYBR FAST qPCR master mix kit (Kapa Biosystems), and the sizes of the libraries were estimated with a 2100 Bioanalyzer (Agilent Genomics) before sequencing on a MiSeq platform using the MiSeq version 3 (600-cycle) reagent kit (Illumina, Inc.).

The quality of the sequences was checked with FastQC version 0.10.1 (http://www.bioinformatics.babraham.ac.uk/projects/fastqc). Cutadapt version 1.3 (7) was used to trim stretches containing unidentified nucleotides (“N”) before quality trimming using Sickle version 1.210 (*Q* score, <30; length, <50 bp) (8). Cell culture contaminants were removed by mapping to *Mycoplasma mycoides*, *M. conjunctivae*, and *M. genitalium* using the Burrows–Wheeler version 0.7.12 alignment tool (9). All reads (2,422,670) were mapped to the closest BLASTn hit (AY640317) using Geneious version 9.1.8 (Biomatter, Ltd.), resulting in a 14,377-bp contig (24,350 reads) with an average coverage of 473×. The protein-coding genes were predicted relative to the reference sequence AY640317 by GATU (10). Phylogenetic analyses of the complete genome confirmed the isolate as avian metapneumovirus subtype A.

### Accession number(s).

The complete genome sequence of aMPV-A/chicken/Brazil-SP/669/2003 has been deposited in GenBank under accession number MF093139.
